# How to interpret a meta-analysis?

**DOI:** 10.1590/1677-5449.202200431

**Published:** 2022-10-03

**Authors:** Regina El Dib

**Affiliations:** 1 Universidade Estadual Paulista – UNESP, Instituto de Ciência e Tecnologia, São Paulo, SP, Brasil.; 2 McMaster University, Departamento de Métodos, Evidências e Impacto de Pesquisa em Saúde – HEI, Hamilton, ON, Canada.

**Keywords:** meta-analysis, systematic review, healthcare

## Abstract

There is an enormous and ever-growing quantity of healthcare information available and practitioners must transform it into knowledge to be able to use it in their clinical practice. Even readers who do not conduct scientific studies themselves need to understand the scientific method in detail to be able to critically evaluate scientific articles. Evidence-based healthcare (EBH) can be defined as the link between good scientific research and clinical practice and systematic reviews constitute one of the forms of research excellence proposed within EBH. Systematic reviews employ rigorous methods that reduce the occurrence of bias. Systematic reviews with meta-analyses generally optimize the results found, because quantitative analysis of the studies included in the review yields additional information. In this paper, we will discuss how to interpret a meta-analysis and how to apply subset and sensitivity analysis strategies and we will also describe possible sources of heterogeneity and common errors that can affect a meta-analysis.

In the 1990s, clinical practice was solely based on studies of pathophysiology, expert opinions, text books, and in vitro and animal studies. However, evidence-based healthcare (EBH) has changed this, facilitating both teaching and research. Nowadays, the combination of rigorous scientific methods, exhaustive literature searches, and clinicians’ experience enable us to base our clinical practice decision-making on high-quality evidence.

This process began with EBH, which can be defined as the link between good scientific research and clinical practice.^1^^,^^2^ In other words, EBH is the application to clinical practice of the results of existing, currently-available scientific proof with good internal and external validity. Alternatively, in 1999, EBH was defined as the conscientious, explicit, and judicious use of the best available research evidence on medical care for patient management.^1^

When we discuss treatment in relation to evidence, we refer to effectiveness, efficacy, efficiency, and safety.^3^ Effectiveness refers to treatment that works in real-life conditions.^3^ Efficacy refers to treatment that works in ideal conditions.^3^ We talk of efficiency when a treatment is cheap and accessible, so that all patients can benefit from it.^3^ Finally, safety means that an intervention has reliable characteristics that make the occurrence of an adverse effect on the patient unlikely.^3^ A study with good internal validity should therefore include the components described above.

The process of EBH starts with construction of a good question of clinical interest. A well-formulated question is the first and most important step for initiating research because it reduces the likelihood of systematic errors (biases) during design, planning, statistical analysis, and conclusion of a research project.^3^ The second step is to identify the best evidence to answer the question of clinical interest – evidence that is pertinent to the epidemiological study design, such as randomized clinical trials (RCTs) and cohort studies.

So, how do we practice EBH? To do so, we need to take the following steps:^3^

1. Transform a need for information into a question that can be answered;
2. Identify the best evidence with which to answer this question (i.e. determine the best study design for the clinical question);
3. Access the most important medical databases, such as Cochrane, PubMed, EMBASE, Web of Science, and LILACS, searching for potentially applicable studies;
4. Conduct a critical analysis of the evidence in terms of its internal validity (i.e., its proximity to the truth), its impact (what is the effect size?), and its applicability to clinical practice;
5. Finally, conduct statistical analyses, including meta-analyses, and interpret the data.

A systematic review (SR) is a type of secondary study design that employs rigorous methods to reduce introduction of biases.^3^^,^^4^ Systematic reviews with meta-analyses generally optimize study results, because quantitative analysis of the studies included in the review yields powerful additional information. Therefore, SRs are currently rated level I evidence for any type of clinical question because they systematically summarize information on a given subject derived from primary studies, such as RCTs, cohort studies, and case-control studies, using reproducible methodology, in addition to integrating information in a critical and unbiased manner to support decision-making and explain the differences and contradictions found in individual studies.

Systematic reviews may or may not include meta-analyses in their results – it depends on the studies they include for review. Meta-analyses are a statistical calculation (i.e. a sum of statistics) applied to the primary studies included in the review^4^ and they increase the statistical power to detect possible differences between study groups and improve the precision of data estimates, thereby narrowing the confidence interval (CI).^4^ Additionally, meta-analyses are also easy to interpret, requiring just a little training and practice.^3^

The fundamental principal underpinning a meta-analysis is increased sample size, which is achieved by analyzing the numerical results of several different studies that have examined the same clinical question, which enables a statistical synthesis of the set of results. To ensure that the results of a meta-analysis have applicable significance, the studies that provide the data for the meta-analysis must be homogenous in terms of clinical and methodological features.

In the field of healthcare, meta-analyses are primarily conducted to make decisions about individual studies that report conflicting results. In these studies, estimates of effect size, such as relative risk (RR) and odds ratios, are dependent on the study design that will be plotted.

The most common method for presenting the results of a meta-analysis is in the form of a forest plot^4^ (Figure 1).^5^ These plots show information from the individual studies and the results of the meta-analysis that summarize the data from the primary studies. In the example shown in Figure 1, the studies included in the meta-analysis are listed in the first column of the meta-analysis, the second and third columns show data from the intervention group of interest and the fourth and fifth columns contain data from the comparative group. In both cases, ENDS and ENNDS, the left-hand column indicates the number of events of the clinical outcome of interest, for example, gingival bleeding, and the right-hand column indicates the total size of the group (Figure 1).

**Figure 1:**
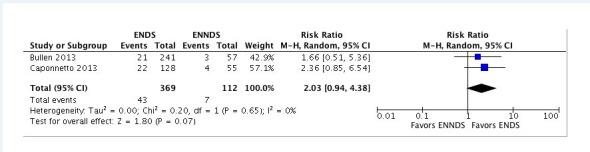
Example of a traditional meta-analysis comparing electronic nicotine delivery systems (ENDS) vs. electronic non-nicotine delivery systems (ENNDS) for smoking cessation.

In this type of plot, each horizontal line represents a primary study included in the meta-analysis with its effect size and respective CI, where the effect size is represented by a square or a circle, depending on the software employed.^4^ The diamond represents the combined results, i.e., the meta-analysis^4^ (Figure 1).

The plot is divided by a vertical line that marks the null effect (RR= 1, or difference between means of 0). If the CI does not contain the null value, i.e. it neither touches nor crosses the vertical line, then the results are considered statistically significant.^4^ When the horizontal line crosses the vertical line (i.e. when the CI does contain the value 1), we can infer that the effect on occurrence of events of the treatment is not significant in the respective study.^4^

A clear example that illustrates the power of meta-analyses is encapsulated in the Cochrane Collaboration logo, which measures the effect size of seven clinical trials that assessed the efficacy of corticosteroids at the end of pregnancy in the mothers of premature babies, where the expected outcome was a reduction in the number of deaths due to pulmonary immaturity^4^ (Figure 2). Only two of the clinical trials exhibited statistically significant effects – i.e. their lines neither touch nor cross the vertical null hypothesis line –, but when the data from all of the studies were groped together, the sample size increased and, consequently, the statistical power improved, indicating that corticosteroids did significantly reduce the risk of babies dying from complications of pulmonary immaturity.^4^

**Figure 2:**
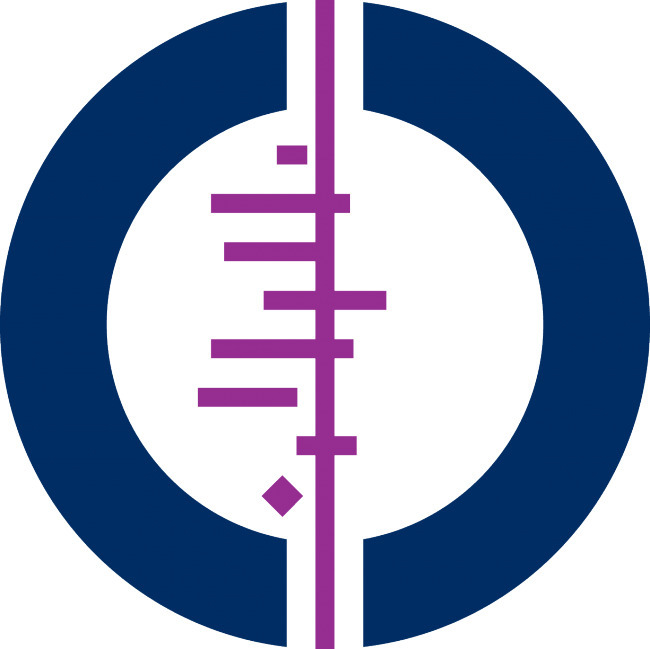
Figure illustrating the Cochrane Collaboration logo.

The advantage of meta-analyses is particularly pertinent in situations in which small studies are being assessed.^4^ Also, when two treatments are compared, the effect size and the differences in results may appear small, but nevertheless be highly relevant. For example, a treatment that reduces mortality from myocardial infarction by 10% in relation to the existing treatment can save the lives of a million people per year.

Larger studies have narrower CIs, i.e. their results are more precise and they make a greater contribution to a meta-analysis, which is also illustrated graphically (the larger the area of the square, the greater the study’s weight in the respective meta-analysis).^4^

The chi-square value is the result of a statistical test of homogeneity of effect size between the studies, i.e., it is a measure of the consistency of the results between the individual studies.^4^ A scale with an I^2^ value close to 0% indicates that the studies are not heterogeneous; values close to 25% indicate low heterogeneity; values close to 50% indicate moderate or substantial heterogeneity; and values close to 75% indicate that the studies are highly heterogeneous.^4^ P values less than 0.1 are considered statistically significant for the heterogeneity calculation, because the I^2^ statistical test has low sensitivity, so the p value threshold is raised to improve sensitivity.^4^ At the bottom of Figure 1, the z value is the result of a statistical test of significance of the overall effect, which is a mathematical measure that is equivalent to the position and width of the diamond in the plot.

There are a number of strategies for investigating heterogeneity when it is statistically significant and thus avoid overestimating the effect of the treatment. One of these strategies is subset analysis.^4^ Subset analysis consists of dividing the sample into two or more subgroups and assessing each one separately.^4^ These subgroups can be separated on the basis of clinical variables. If we take the example of a clinical question from Dentistry, about the effectiveness of phytotherapeutics as adjuvant treatment to scraping and planing the roots for reducing gingival bleeding in patients with periodontitis, we could plan a subset analysis with respect to the control group, i.e., divide the studies included in a meta-analysis according to the control group assessed. For example, chlorhexidine combined with scraping and planing the roots vs. placebo combined with scraping and planing the roots. Moreover, we can employ the same strategy to reduce possible heterogeneity caused by methodological variables, such as analyzing studies judged to have a high risk of bias together with studies classified as at low risk of bias, testing whether the treatment size changes in these subset analyses.

Another strategy for dealing with heterogeneity amongst the studies included in a meta-analysis is to employ sensitivity analysis.^4^ This type of analysis is used to determine the sensitivity of the results of an SR when the methodological premises are modified according to the original plan of action, for example, exclusion of one study from the meta-analysis because its patients had poor prognosis for a specific clinical outcome at the baseline assessment, in contrast with the other studies included in the meta-analysis. After excluding that study, an assessment is made of whether the findings of the primary analysis are confirmed by the findings of the sensitivity analysis and, thus, determine the degree of certainty of the evidence.^4^

It is pertinent to mention that certain errors are common in meta-analyses.^4^ The first such error is “data entry error”, such as, for example, when the mean and standard deviation for the intervention and control groups are inverted.^4^ The second is related to outliers, such as a minus sign omitted from the mean of a continuous outcome or inputting a standard error in place of a standard deviation, also in relation to continuous outcomes.^4^ A third type of error is to include data from a single study several times in the same meta-analysis, thereby overestimating its effect with regard to the treatment in question.^4^ A fourth error is to use the difference between means rather than standardized means for outcomes that employ different assessment tools, as is the case with quality of life assessment questionnaires.^4^ Finally, another possible error is to employ a fixed effects model instead of a random effects model in a meta-analysis with more than two studies, since fixed effects models generally ignore possible heterogeneity between the studies included and therefore overestimate the RR or odds ratio.

Interpretation of a proportional meta-analysis is similar to interpretation of a traditional meta-analysis.^6^ Each horizontal line in the forest plot represents a case series study.^6^ The length of each line corresponds to the study’s 95%CI. The estimated effect size is illustrated with a black square. The size of each square represents the weight of the respective study in the proportional meta-analysis. The combined estimate is shown as an unshaded diamond at the end of the forest plot. The CIs for combined estimates are shown as a horizontal line passing through the diamond.^5^

In this type of statistical analysis, the difference between the interventions studied is considered statistically significant if their respective combined CI estimates do not overlap^6^ (Figures 3, 4 and 5).

**Figure 3:**
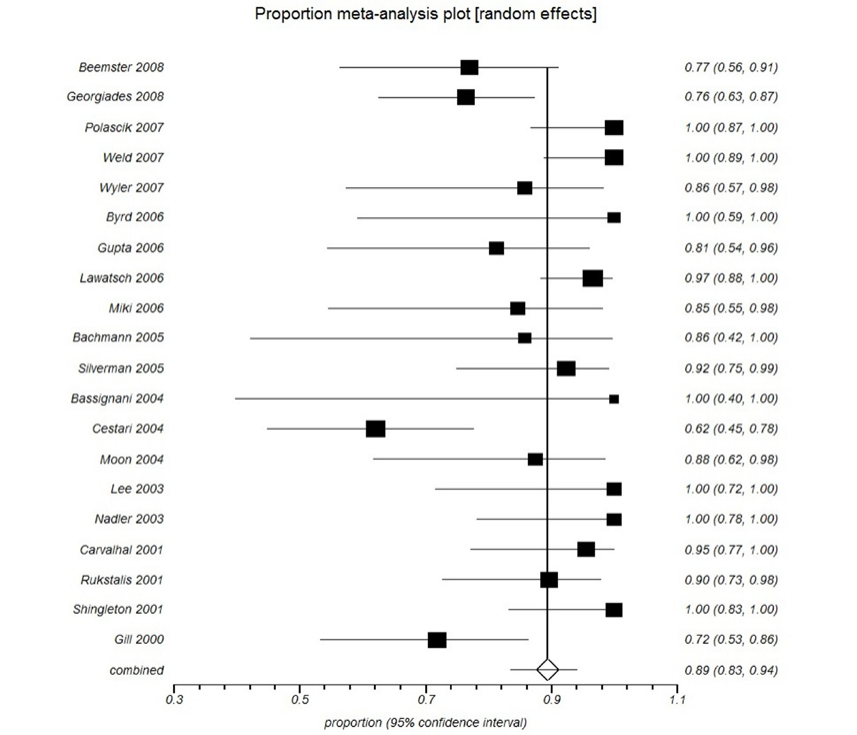
Example of a proportional meta-analysis of cases series on the clinical efficacy of cryoablation.

**Figure 4:**
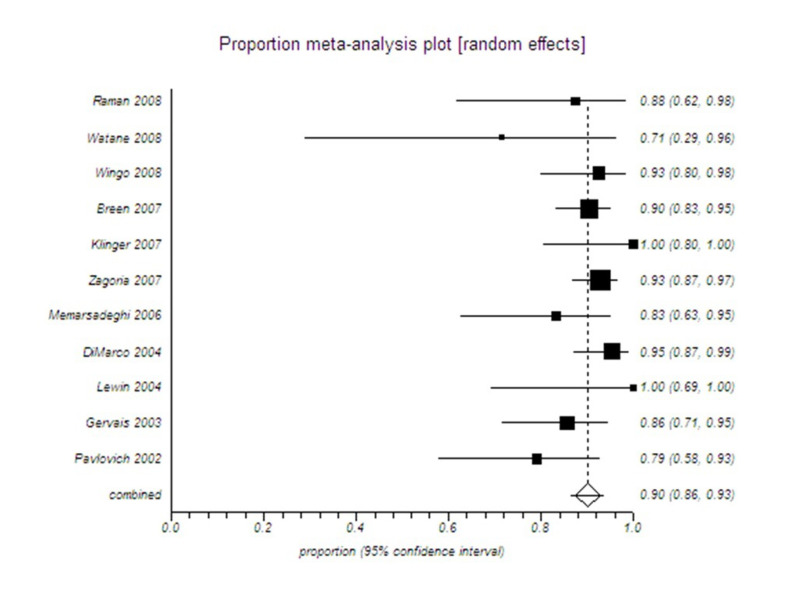
Example of a proportional meta-analysis of cases series on the clinical efficacy of radio frequency ablation

**Figure 5:**
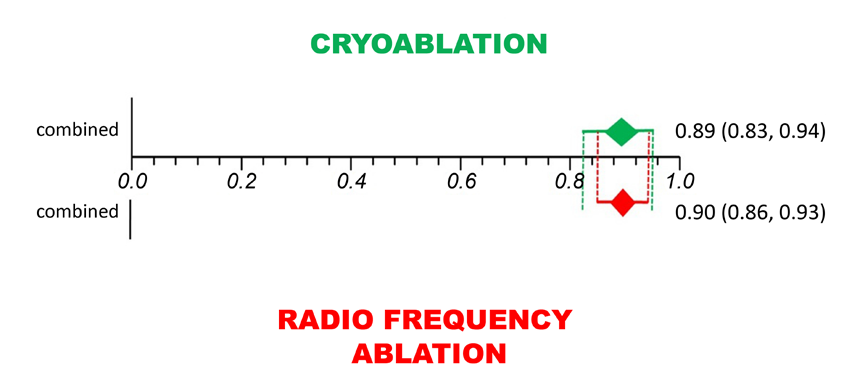
Example of superimposition of the confidence intervals from Figures 3 and 4.

Superimposing the CIs from Figures 3 and 4 will show whether there was in fact a statistically significant difference between the study groups; in this case, between cryoablation and radio frequency ablation (Figure 5). There was no statistically significant difference between cryoablation and radio frequency ablation, since their respective CIs do overlap (Figure 5).

Moreover, whenever possible, a funnel plot should be calculated to evaluate the possibility of publication bias, as a supplement to this statistical analysis. If the funnel plot is asymmetrical, it indicates there is a possibility of publication bias, which is the tendency for scientific journals to publish more articles with positive results than studies with negative results, in addition to a search strategy with low sensitivity and breadth, skewing the results available for testing in the SR.^4^ In this type of meta-analysis, superimposition of the CIs will show whether there is in fact a statistically significant difference between the study groups.^6^^,^^7^

Therefore, clinicians employ SRs with meta-analyses to keep themselves well-informed on certain subjects in medicine; researchers use them to identify, justify, and formulate new hypotheses; healthcare administrators use SRs to formulate clinical guidelines and legislation relating to use of diagnostic tests and assessments of healthcare technology; and, finally, clients base their decisions on the evidence from SRs to reduce their health-related risk.

The advantage of a forest plot is that it summarizes in a single figure all of the information that must be evaluated on the effect and precision of a treatment, thereby increasing sample size and statistical power and detecting a statistically significant difference, if a difference truly exists. Personally, I tend to compare meta-analysis with a quantum leap, in that, in the same way that the fifth dimension enables movements to a parallel future, meta-analysis can anticipate important findings that would otherwise only be identified in the distant future.
