# Supplementary material for: How to interpret a meta-analysis?
Source: J Vasc Bras. 2022 Oct 3;21:e20220043. doi: 10.1590/1677-5449.202200431 (PMC9536318; doi:10.1590/1677-5449.202200431)
Supplement: Translation

# Translation: Como interpretar uma metanálise?

**DOI:** 10.1590/1677-5449.202200432

**Authors:** Regina El Dib

**Affiliations:** 1 Universidade Estadual Paulista – UNESP, Instituto de Ciência e Tecnologia, São Paulo, SP, Brasil.; 2 McMaster University, Departamento de Métodos, Evidências e Impacto de Pesquisa em Saúde – HEI, Hamilton, ON, Canada.

**Keywords:** metanálise, revisão sistemática, saúde

## Abstract

Como a quantidade de informações disponíveis na área da saúde é enorme e crescente, o profissional tem necessidade de transformá-las em conhecimento e utilizá-las em sua prática clínica. Assim, mesmo que o leitor não venha a produzir estudos científicos, é necessário conhecer detalhes da metodologia científica para poder, com espírito crítico, avaliar os artigos científicos. A Saúde Baseada em Evidências (SBE) é definida como o elo entre a boa pesquisa científica e a prática clínica, e uma das pesquisas de excelência propostas pela SBE são as revisões sistemáticas (RSs). As RSs utilizam métodos rigorosos que diminuem a ocorrência de vieses. As RSs com metanálises geralmente otimizam os resultados achados, pois a análise quantitativa dos estudos incluídos na revisão fornece informações adicionais. Neste trabalho, discorreremos sobre como interpretar uma metanálise e como aplicar as estratégias de análises de subgrupo e sensibilidade, além de como descrever possíveis heterogeneidades e erros comuns que acontecem em uma metanálise.

Na década de 1990, a prática clínica era embasada apenas por estudos fisiopatológicos, opiniões de especialistas no assunto, livros-texto e pesquisas *in vitro* e animal. Entretanto, a Saúde Baseada em Evidências (SBE) mudou esse cenário, facilitando o ensino e a pesquisa. Mediante rigorosos métodos científicos e buscas exaustivas pela literatura associada à experiência do clínico, a tomada de decisões baseia-se em evidências de alta qualidade em nossa prática clínica.

Esse processo originou-se com a SBE, sendo definida como o elo entre a boa pesquisa científica e a prática clínica^1^^,^^2^. Em outras palavras, a SBE utiliza provas científicas existentes e disponíveis no momento, com boa validade interna e externa para a aplicação de seus resultados na prática clínica. Outrossim, em 1999, a SBE foi definida como o uso consciencioso, explícito e judicioso da melhor evidência disponível de pesquisa de cuidados médicos na administração dos pacientes^1^.

Quando abordamos o tratamento e falamos em evidências, referimo-nos a efetividade, eficácia, eficiência e segurança^3^. A efetividade diz respeito ao tratamento que funciona em condições de mundo real^3^. A eficácia diz respeito ao tratamento que funciona em condições de mundo ideal^3^. Referimo-nos à eficiência quando o tratamento é barato e acessível, de forma que todos os pacientes possam dele usufruir^3^. Por último, a segurança significa que uma intervenção possui características confiáveis que tornam improvável a ocorrência de algum efeito adverso ao paciente^3^. Portanto, um estudo com boa validade interna deverá apresentar os componentes descritos anteriormente.

O processo da SBE inicia-se pela formulação de uma boa questão clínica de interesse. Uma boa pergunta formulada é o primeiro e mais importante passo para o início de uma pesquisa, pois diminui a possibilidade de erros sistemáticos (vieses) durante a elaboração, o planejamento, a análise estatística e a conclusão de um projeto de pesquisa^3^. O segundo passo é identificar a melhor evidência para responder à questão clínica de interesse – evidências pertinentes ao desenho de estudo epidemiológico, como ensaios clínicos randomizados (ECRs) e estudos coortes.

Então, como praticamos a SBE? Para isso, devemos seguir os seguintes passos^3^:

1. Transformação da necessidade de uma informação em uma pergunta que possa ser respondida;
2. Identificação da melhor evidência com a qual responder a essa pergunta (ou seja, verificação do melhor desenho de estudo de acordo com a questão clínica);
3. Acesso às principais bases de dados da área da saúde como Cochrane, PubMed, EMBASE, Web of Science e LILACS, em busca de estudos em potencial;
4. Realização da avaliação crítica das evidências em relação à validade interna (ou seja, proximidade da verdade), ao impacto (que seria o tamanho do efeito) e à aplicabilidade na prática clínica;
5. Por fim, realização da análise estatística, incluindo as metanálises, e da interpretação dos dados.

As revisões sistemáticas (RSs) são um desenho de estudo secundário que utiliza métodos rigorosos com o intuito de diminuir a ocorrência de vieses^3^^,^^4^. As RSs com metanálises geralmente otimizam os resultados de um estudo, pois a análise quantitativa dos estudos incluídos na revisão fornece informações poderosas e adicionais. Dessa forma, as RSs são consideradas atualmente o nível I de evidências para qualquer questão clínica por sumarizarem sistematicamente informações sobre um determinado tópico por meio de estudos primários, como os ECRs, os estudos coortes e os estudos caso-controle, utilizando-se de uma metodologia reprodutível, além de integrar informações de forma crítica e isenta para auxiliar as decisões e explicar as diferenças e contradições encontradas em estudos individuais.

As RSs podem ou não conter metanálises em seus resultados – isso vai depender dos estudos incluídos. As metanálises são um cálculo estatístico (ou seja, um somatório estatístico) aplicado aos estudos primários incluídos em uma revisão^4^ e aumentam o poder estatístico para detectar as possíveis diferenças entre os grupos estudados e a precisão da estimativa dos dados, diminuindo, assim, o intervalo de confiança (IC)^4^. Além disso, as metanálises são fáceis de serem interpretadas, dependendo apenas de um pouco de prática e treino^3^.

A metanálise tem como princípio fundamental o aumento do tamanho amostral, o qual é obtido pelos resultados numéricos de vários estudos examinando a mesma questão clínica, o que permite a realização de uma síntese estatística do conjunto de resultados. Para que o resultado de uma metanálise tenha significado aplicado, os estudos que compõem os dados da metanálise devem apresentar homogeneidade em relação aos aspectos clínicos e metodológicos.

Na área da saúde, são realizadas metanálises para verificação, principalmente sobre estudos individuais que apresentem resultados conflitantes. Nesses estudos, são apresentadas estimativas para as medidas de efeito, tais como o risco relativo (RR) e a razão de chances, dependendo do desenho de estudo a ser plotado.

A maneira mais usual de apresentar os resultados de uma metanálise é através do gráfico chamado *forest plot*^4^ (Figura 1)^5^. Esse gráfico mostra informações individuais dos estudos e os resultados da metanálise que sumarizam os dados dos estudos primários. No exemplo mostrado na Figura 1, na primeira coluna de uma metanálise, estão listados os estudos que entraram naquela metanálise. A segunda e terceira colunas contêm os dados do grupo da intervenção de interesse, e a quarta e quinta colunas contêm os dados do grupo-controle. Tanto em ENDS quanto em ENNDS, a coluna da esquerda indica o número de eventos do desfecho clínico de interesse, por exemplo, sangramento gengival, e a coluna da direita representa o tamanho total do respectivo grupo (Figura 1).

**Figura 1:** Exemplo de uma metanálise tradicional comparando cigarros eletrônicos com nicotina (ENDS) *versus* cigarros eletrônicos sem nicotina (ENNDS) para cessação do tabagismo.

Nesse tipo de gráfico, cada linha horizontal representa um estudo primário incluído na metanálise com a medida de efeito e seu respectivo IC, sendo que a medida de efeito é representada por um símbolo que pode ser um quadrado ou um círculo, dependendo do *software* utilizado^4^. O losango ou diamante representa a combinação dos resultados, ou seja, a metanálise^4^ (Figura 1).

O gráfico é dividido por uma linha vertical que marca o efeito nulo (o valor igual a 1, no caso do RR, e igual a zero, no caso da diferença de média). Se o IC não contiver o valor neutro, ou seja, não tocar nem cruzar a linha vertical, esses resultados são ditos como estatisticamente significantes^4^. Quando a linha horizontal atravessar a linha vertical (ou seja, quando o IC contiver o valor 1), podemos inferir que, naquele respectivo estudo, o efeito do tratamento sobre a ocorrência do evento não é significativo^4^.

Um exemplo claro para descrever o poder das metanálises é demonstrado no logotipo da Colaboração Cochrane, que mensura o tamanho de efeito de sete ensaios clínicos que avaliaram a eficácia do uso de corticosteroide no final da gravidez de mães de bebês prematuros, sendo esperada como desfecho a diminuição do número de mortes por imaturidade pulmonar^4^ (Figura 2). Apenas dois ensaios clínicos apresentaram efeitos estatisticamente significantes – ou seja, não tocaram nem cruzaram a linha vertical da hipótese nula –, embora, quando agrupados os dados de todos os estudos, o tamanho amostral tenha aumentado e, consequentemente, tenha melhorado o poder estatístico, indicando que o corticosteroide reduz significativamente o risco de bebês morrerem por complicações de imaturidade pulmonar^4^.

**Figura 2:** Figura ilustrativa do logotipo da Colaboração Cochrane.

A vantagem das metanálises é particularmente pertinente também em condições de avaliações de estudos pequenos^4^. Além disso, quando se comparam dois tratamentos, o efeito e as diferenças de resultados podem se apresentar pequenos, porém muito relevantes. Por exemplo, um tratamento que reduz a mortalidade por infarto do miocárdio em 10% em relação ao tratamento já existente pode salvar milhões de pessoas por ano.

Os estudos maiores possuem ICs mais estreitos, ou seja, resultados mais precisos e maior contribuição para a metanálise, que também é representada graficamente (quanto maior a área do quadrado, maior o peso do estudo nessa respectiva metanálise)^4^.

O valor do qui-quadrado é um teste estatístico de homogeneidade do tamanho do efeito entre os estudos, isto é, uma medida da consistência do resultado entre os estudos individuais^4^. A escala com valor de I^2^ próximo a 0% indica não heterogeneidade entre os estudos; com valor próximo de 25% indica baixa heterogeneidade; com valor próximo de 50% indica heterogeneidade moderada ou substancial; e com valor próximo de 75% indica alta heterogeneidade^4^. Considera-se o valor de p menor que 0,1 como estatisticamente significante para o cálculo de heterogeneidade, pois o teste estatístico I^2^ possui baixa sensibilidade, então aumenta-se o *threshold* do valor de p para aumentar a sua sensibilidade^4^. Na parte de baixo do gráfico, o valor de z é um teste estatístico da significância do efeito global, sendo uma medida matemática equivalente à localização e à largura do diamante no gráfico.

Existem algumas estratégias para explorarmos a heterogeneidade quando estatisticamente significante e evitarmos, assim, uma superestimativa no efeito do tratamento. Uma dessas estratégias é a análise de subgrupo^4^. A análise de subgrupo consiste em dividir a amostra em dois ou mais grupos e avaliar separadamente cada um desses subgrupos^4^. A separação desses grupos pode ser feita a partir de variáveis clínicas. Se tomarmos como exemplo a questão clínica na área da Odontologia sobre a efetividade dos fitoterápicos como adjuvantes à raspagem e ao alisamento radicular na redução do sangramento gengival de pacientes com periodontite, então poderíamos planejar uma análise de subgrupo referente ao grupo-controle, ou seja, dividir os estudos incluídos de uma metanálise de acordo com o grupo-controle avaliado, por exemplo, clorexidina associada à raspagem e ao alisamento radicular *versus* placebo associado à raspagem e ao alisamento radicular. Além disso, podemos lançar mão dessa mesma estratégia para diminuir a possível heterogeneidade referente às variáveis metodológicas, como avaliar os estudos julgados como alto risco de viés com os estudos classificados como baixo risco de viés e verificar se a magnitude do tratamento modifica de acordo com essas análises de subgrupo.

Outra estratégia para lidar com a heterogeneidade dos estudos incluídos em uma metanálise é lançarmos mão da análise de sensibilidade^4^. Essa análise é utilizada para determinar a sensibilidade dos resultados de uma RS quando as premissas metodológicas são modificadas de acordo com o plano de ação original, por exemplo, a exclusão de um estudo da metanálise devido aos pacientes apresentarem um prognóstico ruim na avaliação inicial para um determinado desfecho clínico, destoando dos demais estudos incluídos na metanálise. Após a exclusão do estudo, deve-se avaliar se os achados da análise primária corroboram ou não com os achados da análise de sensibilidade e, assim, determinar a certeza das evidências^4^.

Vale ressaltar que existem alguns erros comuns que acontecem em uma metanálise^4^. O primeiro desses erros diz respeito ao “erro de entrada de dados”, ou seja, quando existe, por exemplo, a troca de média e desvio padrão entre os grupos de intervenção e grupo-controle^4^. O segundo erro diz respeito a um *outlier*, como um sinal de menos deixado de fora do valor de uma média de um desfecho contínuo ou a entrada de um erro padrão em vez do valor de um desvio padrão, também em se tratando de um desfecho contínuo^4^. Um terceiro equívoco seria incluir os dados de um mesmo estudo várias vezes em uma mesma metanálise, promovendo, assim, uma superestimativa do efeito daquele tratamento em questão^4^. Uma quarta falha seria considerar a diferença de média em vez da média padronizada para os desfechos que apresentem diferentes ferramentas de avaliação, como é o caso dos questionários para avaliar a qualidade de vida^4^. Por último, outro erro seria considerar um modelo de efeito fixo no lugar do modelo de feito randômico para uma metanálise com mais de dois estudos incluídos; modelos de efeito fixo geralmente ignoram possível heterogeneidade entre os estudos incluídos em uma metanálise e, portanto, superestimam o valor do RR ou da razão de chances.

A interpretação de uma metanálise proporcional é semelhante a uma metanálise tradicional^6^. Cada linha horizontal em um gráfico de floresta representa um estudo incluído do tipo série de casos^6^. O comprimento de cada linha corresponde ao IC de 95%. A estimativa do efeito é marcada com um quadrado preto. O tamanho do quadrado representa o peso que o respectivo estudo exerce na metanálise proporcional. A estimativa combinada é marcada com um losango ou diamante não preenchido no final do gráfico de floresta. Os ICs de estimativas combinadas são mostrados com uma linha horizontal cortando o diamante ou losango^6^.

Nessa análise estatística, consideramos estatisticamente significante uma diferença entre as intervenções estudadas se os respectivos ICs de estimativas combinadas não se sobrepuserem^6^ (Figuras 3, 4 e 5).

**Figura 3:** Exemplo de uma metanálise proporcional de séries de casos sobre a eficácia clínica da crioablação.

**Figura 4:** Exemplo de uma metanálise proporcional de séries de casos sobre a eficácia clínica da ablação por radiofrequência.

**Figura 5:** Exemplo de sobreposição dos intervalos de confiança das Figuras 3 e 4.

As sobreposições dos ICs das Figuras 3 e 4 mostrarão de fato se houve uma diferença estatisticamente significante entre os grupos estudados; nesse caso, entre a crioablação e a ablação por radiofrequência (Figura 5). Não houve diferença estatisticamente significante entre a crioablação e a ablação por radiofrequência, pois seus respectivos ICs se sobrepuseram (Figura 5).

Além disso, quando possível, calcula-se o gráfico de funil para avaliar a possibilidade de viés de publicação como complemento dessa análise estatística. Quando o gráfico de funil for assimétrico, ele sinaliza a possibilidade de viés de publicação, que é a tendência de as revistas científicas publicarem mais artigos com resultados positivos do que com resultados negativos, além de uma estratégia de busca pouco sensível e abrangente, tornando tendenciosos os resultados disponíveis para confronto em uma RS^4^. Nesse tipo de metanálise, a sobreposição dos ICs mostrará se houve de fato uma diferença estatisticamente significante entre os grupos estudados^6^^,^^7^.

Dessa forma, os clínicos utilizam as RSs com metanálises para se manterem bem informados em determinados assuntos na área medica; os pesquisadores usufruem delas para identificar, justificar e formular mais hipóteses; os administradores da saúde usam as RSs para formular diretrizes clínicas e legislação no que concerne ao uso de testes diagnósticos e avaliações tecnológicas em saúde; e, por fim, os consumidores baseiam suas decisões nas evidências existentes de uma RS para reduzirem os riscos relacionados à saúde.

A vantagem do *forest plot*, ou gráfico de floresta, é sumarizar, em uma única figura, todas as informações sobre o efeito e a precisão do tratamento a ser avaliado, aumentando, dessa forma, o tamanho amostral e o poder estatístico e detectando, assim, uma diferença estatisticamente significante, se essa diferença for real. Particularmente, eu costumo comparar a metanálise com um salto quântico, em que, da mesma forma que a quinta dimensão nos possibilita movimentarmos para um futuro paralelo, a metanálise pode antecipar achados importantes que só seriam identificados em um futuro mais distante.
